# Systematic review of assessment instruments measuring outcomes in psychological interventions for pediatric functional neurological disorders

**DOI:** 10.1093/jpepsy/jsaf071

**Published:** 2025-08-25

**Authors:** Barbara Zuro Jakovac, David Hevey, Phillip Coey, Clare Harris, Gary Byrne

**Affiliations:** Department of Psychology, Trinity College Dublin, Dublin, Ireland; Department of Psychology, Trinity College Dublin, Dublin, Ireland; Department of Psychology, Trinity College Dublin, Dublin, Ireland; Children’s Health Ireland, Pediatric Psychology Department, Dublin, Ireland; Department of Psychology, Trinity College Dublin, Dublin, Ireland; Health Service Executive, Primary Care Services, Dublin, Ireland

**Keywords:** functional neurological disorder, psychosocial intervention, measure development and validation, school-age children, adolescents

## Abstract

**Objective:**

This systematic review focused on identifying and evaluating assessment tools used to measure outcomes of psychological interventions for pediatric functional neurological disorder (FND).

**Method:**

A comprehensive search was conducted on September 24, 2024, across Web of Science, PsycINFO, and Medline. Studies were included if they involved individuals under 18 with FND diagnosis, utilized a psychological intervention, and assessed treatment outcomes using validated measures. Sixteen studies qualified for inclusion, and 26 different assessment instruments were identified. These were assessed against the Core Outcome Measures in Effectiveness Trials framework, covering symptoms, life impact, and resource utilization. The psychometric characteristics of these assessment tools were examined through further searches which concentrated on reliability, validity, and factorial invariance. The Joanna Briggs Institute critical appraisal tools were used to evaluate the risk of bias. Findings were synthesized narratively due to the descriptive and exploratory nature of the research aims.

**Results:**

No outcome assessment tools designed specifically for pediatric FND populations were identified. Most studies employed measures targeting mental health symptoms and life impact, however, none of these tools were validated with FND samples, and several lacked validation in pediatric populations.

**Conclusions:**

This review highlights significant gaps, including the need for psychometric assessment of tools and validation studies on FND samples. The current evidence does not support recommending FND-specific measures due to their limited development and validation. Instead, using existing questionnaires validated on broader pediatric populations is recommended. Research should prioritize the validation of measures in FND populations to establish more robust, standardized tools for clinical and research use.

Functional neurological disorder (FND) is a complex condition that involves multiple brain networks and often results in various neurological symptoms (Perez et al., 2021; Vassilopoulos et al., 2022). It is characterized by motor and sensory symptoms, such as limb weakness, paralysis, tremors, nonepileptic seizures (NES), and sensory loss. These symptoms show patterns that do not align with recognized neurological or medical conditions and are not better explained by any other disorder. FND symptoms are associated with considerable distress and impairment in important areas of daily functioning (American Psychiatric Association, 2022). Though research remains limited, there has been an increase of studies investigating intervention methods for this population. Analyzing the tools used to determine intervention outcomes and their validity and reliability could allow for better comparison of various treatments used for FND.

Identifying suitable outcome measures for FND is challenging for several reasons. Firstly, FND can present with numerous symptoms (Martino et al., 2017) that can vary significantly in severity and progression, and fluctuate over time and in different contexts (Mavroudis et al., 2024). Additionally, many children with FND exhibit accompanying functional somatic symptoms, such as pain and fatigue, and psychological comorbidities including anxiety, depression, and other mental health disorders (Vassilopoulos et al., 2022). Recently, there has been a shift toward a holistic understanding of FND, with the biopsychosocial model offering an overarching framework to guide assessment and intervention (Joos et al., 2023; Kola & LaFaver, 2022). This model considers an interplay of biological, psychological, and social factors that shape clinical presentation and treatment needs (Vassilopoulos et al., 2022). Clinicians often need to consider these factors in interventions and target various areas, such as maladaptive behaviors and cognitions, sleep routines, emotion regulation, and family dynamics (Fobian et al., 2020; Kozlowska et al., 2021; Shafir, 2016; Vassilopoulos et al., 2022). All these factors suggest a range of potentially important outcomes to target.

The importance of consistent outcome measurement has been highlighted in health research (Pick et al., 2020). The Core Outcome Measures in Effectiveness Trials (COMET) initiative in Europe offers guidance about core outcome sets that should be measured and reported in research, clinical audits, and routine care. While outcome represents *what* is being measured, core outcome sets provide a standardized list of outcomes that should be assessed in research of a specific illness or diagnosis. The aim is to gather data that would allow examination and comparison of results across studies in various areas of health (COMET Initiative, 2018; Prinsen et al., 2014). COMET recommends five important outcome domains: core physiological/clinical symptoms, life impact, resource utilization, adverse events, and mortality. Physiological/clinical symptoms refer to various signs and symptoms of illnesses, which can be further classified based on the underlying cause or system involved. The life impact domain includes measures of person’s functioning, quality of life, subjective health perceptions, personal circumstances, and certain elements of care delivery that shape individual experiences (such as emotional satisfaction with care or accessibility of care). Resource utilization refers to the use and cost of economic, healthcare, and societal resources, including hospital care, the need for further medical interventions, or support and care provided by others. Adverse events are defined as unintended consequences, and mortality refers to survival/mortality outcomes of an intervention.

A previously conducted systematic review identified existing outcome measures for adults with FND (Pick et al., 2020). This study found a lack of validated FND-specific measures in adults. FND symptom change was the most commonly used strategy for measuring outcomes. Other domains captured psychological symptoms, physical symptoms, life impact, and health economics. Due to differences in cognition and communication between adults and children, considerable care is needed in designing and choosing instruments separately for children (Bell, 2007).

Currently, there are no established recommendations for core outcome measure sets in pediatric FND. This study aims to review the literature on assessment tools used for psychological treatment outcomes in pediatric FND and to critically appraise included studies. The COMET framework was applied to examine the extent to which identified instruments captured key outcome domains. Additionally, this study provides insights into the existing evidence related to the psychometric properties of these tools.

## Methods

### Search strategy

A comprehensive search was conducted using EBSCOhost across Web of Science, PsycINFO, and Medline on September 24, 2024. The search terms detailed in Box 1 were applied to abstracts, keywords, and titles. Additional papers were identified using a descendancy approach, which included screening references by the lead author. No timeframe criteria were used, and the year of publication ranged from 1913 to 2024. This review was conducted in accordance with the Preferred Reporting Items for Systematic Reviews (PRISMA) checklist (Page et al., 2021), which is available as Supplementary File S1.

**Box 1.** Search terms.

Functional Neurological Disorder	“Functional Neurological Disorder*” OR FND OR “Conversion Disorder*” OR “Psychogenic Disorder*” OR “Somatoform Disorder*” OR “Nonorganic Neurological Disorder*” OR “Functional Movement Disorder*” OR “Functional Motor Disorder*” OR FMD OR “Psychosomatic Disorder*” OR “Medically Unexplained Disorder*”OR “Medically Unexplained Symptom*” OR MUS OR “Functional Neurological Symptom Disorder*” OR FNSD OR “Hysterical Neuros*” OR “Hysterical seizure*” OR “ OR “Nonepileptic” *Recommended thesaurus concept*—“Conversion disorder”
Pediatric	“paediatric” OR “child*” OR “adolescen*” OR “young people” OR “young person” OR “under 18’s” OR “under 18 years old”
Treatment	“treatment *” OR “intervention*” OR “therap*” OR “training*” OR “ psychotherapy*” OR “counselling” OR “counseling” *Recommended thesaurus concept*—“Child counselling” OR “Child psychotherapy” OR “Adolescent counselling” OR “Adolescent psychotherapy”

### Eligibility criteria

Articles written in English and published in peer-reviewed journals were included. Given the limited research on pediatric treatment outcomes, various study methodologies were considered, including case studies, cohort studies, controlled and uncontrolled studies, and cross-sectional and longitudinal designs. The inclusion criteria were as follows:

1. Participants met the criteria for FND outlined in the Diagnostic and Statistical Manual of Mental Disorders (DSM) or the International Classification of Diseases (ICD) relevant at the time of publication, or they had a confirmed diagnosis of FND following an assessment involving a neurologist (Tolchin et al., 2021). In case of functional neurological seizures, diagnosis could be made through EEG monitoring (LaFrance et al., 2013). Studies were eligible if FND was a primary diagnosis, even if participants had multiple diagnoses. Studies involving various diagnoses were included if it was clear which measures were used with FND participants.
2. The sample consisted of individuals under the age of 18 years. Studies with adults were included if measures used on participants under 18 were clearly identified. The age limit of 18 years was chosen because instruments for children often use this cutoff in the development and validation process.
3. Studies involved a psychological intervention based on a psychological theory or model aimed at modifying psychological processes underlying pain, distress, or disability (Carlson & Nicholson Perry, 2017). This included psychological therapies specified by the UK Department of Health (British Psychological Society Centre for Outcomes Research and Effectiveness, 2001) and third-wave therapies (Perkins et al., 2023). Studies involving only psychoeducation were excluded.
4. Studies used an assessment instrument to measure a construct related to outcomes of the psychological intervention. Instruments needed to be validated, with published analyses demonstrating reliability (e.g., internal consistency, test–retest reliability, interrater reliability) and validity, including construct or criterion validity (Evers et al., 2013).

### Data collection process and risk of bias

The lead author screened titles and abstracts to exclude ineligible studies and duplicates were removed with Covidence. Full texts were independently reviewed by the lead author and a second rater. Any disagreements were resolved through consultation with a third rater. The risk of bias assessment was conducted by the lead author using the Joanna Briggs Institute critical appraisal tools (Munn et al., 2023), which provides a set of tools that can accommodate a wide range of study designs included in the review.

A narrative synthesis approach was chosen for synthesizing findings due to the exploratory and descriptive nature of the research aims. Data extraction was completed by the lead author using the population, interventions, comparison, and outcomes (PICO) framework, which provides a structured and consistent organization of data (Higgins et al., 2024). The identified instruments were then categorized into domains aligned with the COMET framework and summarized in a narrative synthesis by the lead author. The framework for this study was selected based on a previously published systematic review that addressed FND outcome measurement in adult FND (Pick et al., 2020).

## Results

### Study selection

**Figure 1.**
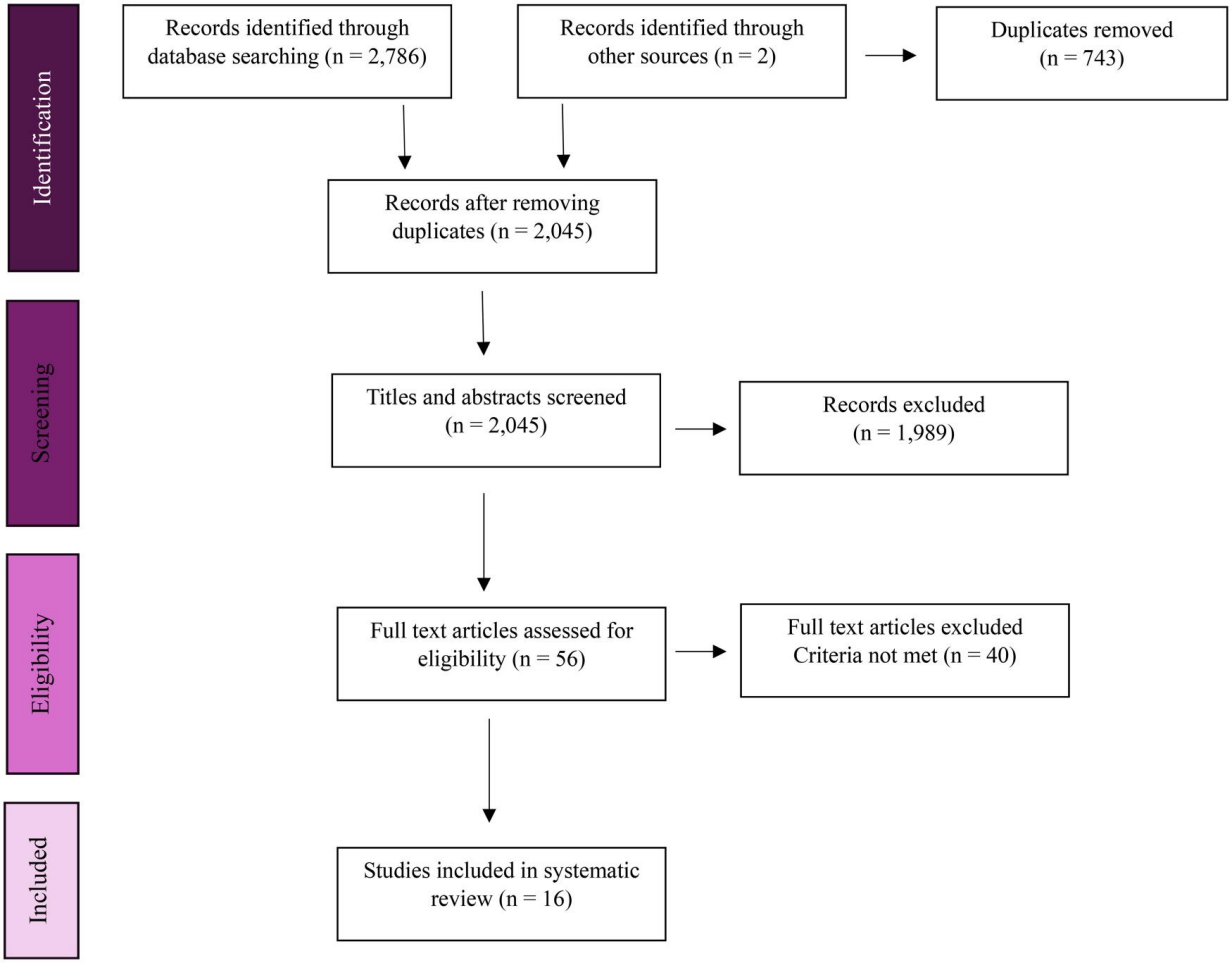
PRISMA flow diagram showing study selection. Identification: 2,786 records from databases and 2 from other sources, 743 duplicates removed. Screening: 2,045 records reviewed, 1,989 excluded. Eligibility: 56 full texts assessed, 40 excluded. Included: 16 studies in final review.

Figure 1 shows the study selection process. A search of three databases produced a total of 2,788 studies. Of these, 743 duplicates were removed. Additionally, two studies were identified through citation searches. Titles and abstracts of 2,045 studies were screened based on inclusion and exclusion criteria. The full texts of 56 studies were assessed for eligibility. In total, 16 studies were included in the final review.

### Study characteristics

Table 1 provides a summary of the 16 included studies. The studies were published between 2014 and 2024. Most studies were case studies and noncontrolled studies, with only one nonrandomized and one randomized controlled trial (RCT). The studies were predominantly conducted in high-income countries, including the USA, Australia, Canada, Ireland, the UK, and Israel. One study was conducted in Iran and one in Turkey. Eleven out of 16 studies had a predominance of adolescent female participants, with ages ranging from 6 to 18 years old. Table 1 also includes timelines for outcome assessment. All studies assessed constructs of interest at a preintervention time point, including inpatient admissions and admissions to an outpatient programme. Posttreatment measurement varied from discharge to a 1-year follow-up. Moreover, five studies included multiple time points for measuring outcomes. For example, a study conducted by Byrne and Connon (2023) measured outcomes at 3- and 5-month follow-up and found some differences in results. This suggests that including multiple time points could provide new information and informed sustainability of outcomes.

**Table 1.** Characteristics of studies meeting inclusion criteria (*N* = 16).

Study	Design	*N*	Population	Intervention	Comparison	Instrument	Preintervention measurement time	Postintervention measurement time	JBI score
Barak et al., 2022	Noncontrolled study	31	Mixed sample, age range 13–16 years, FND	Multidisciplinary inpatient program, including individual and parent psychological therapy	Before and after scores	OMNI Perceived Exertion Scale	Admission	Discharge	6/7
Byrne & Connon, 2023	Case study	1	Female, 12, NES	Acceptance and commitment therapy	Before and after scores	AAQ-II, BYI-II, WSAS-Y	Initial assessment	Three- and five-month follow-up	7/8
Demirci & Sagaltici, 2021	Case studies	2	Female, 13 Male, 16, NES	Eye movement desensitization and reprocessing treatment	Before and after scores	ADES	Initial assessment	Six-month follow-up	7/8
Fobian et al., 2020	RCT	29	Mixed sample, age range 12–17 years, NES	Retraining and Control Therapy	Before and after scores and supportive therapy	A-COPE, BASC-2, CSSI-24, PedsQL	Seven days preintervention	Seven days postintervention and 60 days postintervention	10/13
Howlett et al., 2022	Noncontrolled study	20	Mixed sample, age range 13–15 years, functional tics	Cognitive-behavioral therapy and behavioral interventions	Before and after scores	YGTSS	Initial assessment	Six-month follow-up	5/7
Kempert, 2021	Case study	1	Female, 13, FND and complex regional pain	Multidisciplinary inpatient program, including individual and group psychological treatment	Before and after scores	COMP, LEFS, UEFI	Admission	Four-week discharge and 10-week follow-up	7/8
Klineberg et al., 2014	Noncontrolled study	50	Mixed sample, age range 12–17 years, somatoform disorders, including FND	Multidisciplinary treatment in clinic, including individual counselling/cognitive behavioral therapy	Before and after scores	CHQ	Initial assessment	Four months into treatment and 12 months into treatment	5/7
Kozlowska et al., 2018	Noncontrolled study	60	Mixed sample, age range 8–17 years, NES	Mind Body Programme, including individual and family psychological therapy	Before and after scores	RAHC-GAF	Initial assessment	Minimum 12-month follow-up	8/9
Landa et al., 2023	Nonrandomized controlled trial (retrospective)	31	Mixed sample, age range 6–18 years, FND	Integrative rehabilitation program, including individual and family psychological therapy	Before and after scores and healthy control groups	OMNI Perceived Exertion	Admission	Discharge	8/9
Maxwell et al., 2023	Case series (retrospective)	8	Mixed sample, age range 13–16 years, functional tics	Integrated cognitive behavioral intervention	Before and after scores	YGTSS	Preintervention (unspecified)	Postintervention (unspecified)	10/10
McFarlane et al., 2019	Case series	22	Mixed sample, age range 6–17 years, FND	Family-based cognitive behavior therapy	Before and after scores	CGAS, RCADS, SDQ	Initial assessment	Postintervention (unspecified)	9/10
Nasiri et al., 2015	Case study	1	Male, 12, functional myopia	Cognitive analytical therapy	Before and after scores	BDI-II, SAS	Preintervention (unspecified)	Five-month follow-up	4/8
Rajabalee et al., 2022	Case study	1	Female, 10, FND	Mind-Body Rehabilitation Program with psychological therapy	Before and after scores	BPQ, DASS	Initial assessment	One-month follow-up	7/8
Robinson et al., 2020	Case series	18	Mixed sample, age range 10–18 years, functional movement disorder	Cognitive-behavioral therapy with attention training	Before and after scores	CGAS	Initial assessment	Clinical discussion at the end of treatment	9/10
Scheurich et al., 2024	Noncontrolled study	1	Mixed sample, 13–23, NES	Multidisciplinary inpatient treatment, involving psychological support for identifying early warning signs and active coping	Before and after scores	CES-DC, FDI, PCS-C, SCAS	Admission	Discharge	5/7
Stager et al., 2023	Part of a larger RCT	14	Mixed sample, age range 12–18 years, NES	Retraining and Control Therapy	Before and after scores	BASC-2, CSSI-24, PedsQL	Seven days preintervention	Seven days postintervention and 60 days postintervention	5/7

*Note*. *N*=sample size; JBI=Joanna Briggs Institute critical appraisal score; AAQ-II=Acceptance and Action Questionnaire II; A-COPE=Adolescent Coping Orientation for Problem Experiences; ADES=Adolescent Dissociative Experiences Scale; RCT=randomized controlled trial; BASC-2=Behaviour Assessment System for Children, Second Edition); BDI-II=Beck’s Depression Inventory; FND=functional neurological disorder; NES=nonepileptic seizures; BPQ=Body Perception Questionnaire; CES-DC=Center for Epidemiological Studies—Depression Scale for Children; CGAS=Children’s Global Assessment Scale; COPM=Canadian Occupational Performance Measure; CSSI-24=Children’s Somatic Symptoms Inventory; CHQ=Child Health Questionnaire; DASS=Depression, Anxiety, and Stress Scales; FDI=Functional Disability Inventory; LEFS=Lower Extremity Functioning Scale; PCS-C=Pain Catastrophizing Scale for Children; PedsQL=Pediatric Quality of Life Inventory; RCADS=Revised Child Anxiety and Depression Scale; RAHC-GAF=Royal Alexandra Hospital for Children Global Assessment of Functioning; SCAS=Spence Children’s Anxiety Scale; SDQ=Strengths and Difficulties Questionnaire; UEFI=Upper Extremity Functioning Scale; WSAS-Y=Work and Social Adjustment Scale—Youth Version; YGTSS=Yale Global Tic Severity Scale; SAS=Zung’s Self-rating Anxiety Scale.

### Critical appraisal of studies

The JBI Critical Appraisal Tools assess multiple domains, including clear information about participants and inclusion/exclusion, intervention measurement and clarity, measurement quality, and statistical analysis. Questions were scored as a yes, no, unclear, or not applicable. Yes answers were provided with 1 point, while no and unclear were scored as 0. The answer to each question was summarized to provide a total score, excluding not applicable items (Vickers et al., 2025). Each study was evaluated by the lead author and a co-author, and the interclass correlation coefficient was calculated as a measure of inter-rater reliability. Inter-rater reliability was *ICC*=.95, indicating a high level of agreement between the raters. Observed strengths in the studies included clear descriptions of participants’ backgrounds and clinical information, a clear outline of interventions, and consistency in pre- and postmeasurement. Common weaknesses among studies included small sample sizes, lack of control groups, and failure to address adverse events. While all included studies used at least one measure validated on child samples, none of the measures used have been validated with children with FND. Details of the quality appraisal criteria are provided in the Supplementary Materials.

### Assessment instruments in FND intervention studies

Twenty-six different instruments were identified. All instruments have been used as measures of outcomes following an intervention. However, while some instruments captured symptoms of FND, many examined comorbid difficulties often seen in FND populations (Vassilopoulos et al., 2022). There was some consistency in the outcome domains measured, but a variety of instruments were employed across studies. Each instrument was evaluated to determine how it aligned with the key domains in the COMET framework, including symptoms, life impact, and resource utilization. Many studies assessed functional independence and quality of life, which fit with the COMET life impact category as they reflect individuals’ ability to engage in daily activities. Given the range and variability of symptoms measured, they were further classified into core FND symptoms, mental health, and other symptoms (Pick et al., 2020). Core FND domain included motor or sensory symptoms not explained by recognized medical or neurological conditions. Mental health symptoms refer to psychological difficulties that impact person’s thoughts, emotions, or behaviors. Finally, the other symptoms category was created to encompass symptoms that do not fall within these two categories, but can present in children with FND, such as pain, fatigue, or high exertion.

The domains of mortality and adverse events were excluded, as the instruments were not designed to assess these outcomes. While some instruments or items might provide insight into the unintended consequences of interventions, they were not used for this purpose in the included studies.

#### COMET domains

##### Core FND symptoms

Only two studies used an instrument for measuring core symptoms of FND. Howlett et al. (2022) and Maxwell et al. (2023) utilized the Yale Global Tic Severity Scale (YGTSS; Leckman et al., 1989), which is a semistructured clinical interview for assessing the severity of tics. The YGTSS measures number, frequency, intensity, complexity, and overall impact of motor and vocal tics. The YGTSS also includes items that assess the impact of tics on communication, daily activities, family, relationships, and school, which can provide insights into child’s overall functioning.

##### Mental health

Seven studies used questionnaires for depression and/or anxiety (Byrne & Connon, 2023; Fobian et al., 2020; McFarlane et al., 2019; Nasiri et al., 2015; Rajabalee et al., 2022; Robinson et al., 2020; Scheurich et al., 2024; Stager et al., 2023). Tools included the Beck’s Youth Inventory (BYI-II) and Beck’s Depression Inventory (BDI-II; Beck et al., 1996), Depression Anxiety Stress Scale (DASS; Lovibond & Lovibond, 1995), Center for Epidemiological Studies—Depression Scale for Children (CES-DC; Weissman et al., 1980), Spence Children’s Anxiety Scale (SCAS; Spence et al., 2003), Zung Self-Rating Anxiety Scale (SAS; Zung, 1971), Revised Child Anxiety and Depression Scale (RCADS; Chorpita et al., 2000), and Behaviour Assessment System for Children (BASC-2; Reynolds & Kamphaus, 2004). While RCADS can evaluate a broad range of symptoms, only the total anxiety and depression subscales were reported in one study (McFarlane et al., 2019). Moreover, only some of the BASC-2 scales were employed. Stager et al. (2023) used self-report measures for anxiety, depression, and social stress, while Fobian et al. (2020) reported on measures of anxiety and depression.

McFarlane et al. (2019) used the Strengths and Difficulties Questionnaire (SDQ; Goodman, 1997), which is a multidimensional parent and child measure of emotional symptoms, conduct problems, hyperactivity, peer relationship difficulties, and prosocial behavior. Other psychological symptoms that were measured were dissociative experiences, psychological flexibility, and catastrophic thinking. One study (Demirci & Sagaltici, 2021) measured dissociative symptoms by employing the Adolescent Dissociative Experiences Scale (A-DES; Armstrong et al., 1997). Byrne and Connon (2023) used the Acceptance and Action Questionnaire II (AAQ-II; Bond et al., 2011) as a measure of psychological flexibility. Lastly, the Pain Catastrophizing Scale for Children (PCS-C; Crombez et al., 2003) was used in one study (Scheurich et al., 2024) to assess catastrophic thoughts related to pain.

##### Other symptoms

Four studies included measures of other symptoms. Two studies used (Barak et al., 2022; Landa et al., 2023) the OMNI Perceived Exertion Scale (OMNI-RPE; Robertson et al., 2000) to assess subjective intensity of physical effort. Rajabalee et al. (2022) used the Body Perception Questionnaire (BPQ; Porges, 1993) to examine frequency of body stress reactions. Additionally, Stager et al. (2023) measured somatic symptoms using the BASC somatic subscale and the Children’s Somatic Symptoms Inventory-24 (CSSI-24; Walker et al., 2009).

##### Life impact

Life impact was assessed in nine studies. The Functional Disability Inventory (FDI; Walker et al., 1991) was used to assess how a child’s physical health impacted their psychosocial functioning and daily activities in one study (Scheurich et al., 2024). Byrne & Connon (2023) utilized the Work and Social Adjustment Scale-Youth Version (WSAS-Y; Jassi et al., 2020), a self-reported measure that evaluated the impact of symptoms on functioning in school and socialization. The Canadian Occupational Performance Measure (COPM; Law et al., 2019) was used as a semistructured interview (Kempert, 2021) to assess self-care, productivity, and leisure. This study also used the Lower Extremity Functional Scale (LEFS; Binkley et al., 1999) and Upper Extremity Functional Index (UEFI; Stratford et al., 2001), which measured the difficulty individuals faced in performing daily activities due to limb impairments.

Additionally, two studies (McFarlane et al., 2019; Robinson et al., 2020) applied the Children’s Global Assessment Scale (CGAS; Shaffer et al., 1983), a clinician-rated measure assessing functioning across psychological, social, and academic domains. Another study (Kozlowska et al., 2018) used a different version of the CGAS, the Royal Alexandra Hospital for Children Global Assessment of Functioning, which broadens the concept of pediatric health status (RAHC-GAF; Dossetor et al., 1996). Two studies (Fobian et al., 2020; Stager et al., 2023) employed the Pediatric Quality of Life Inventory (PedsQL; Varni et al., 1999), an instrument designed to measure health-related quality of life from both parent and self-report perspectives, however, only the self-reported version was used in these studies. The PedsQL can compute four core scales, providing information about person’s physical, emotional, social, and school functioning. Lastly, one study (Klineberg et al., 2014) used both self- (CHQ-CF) and parent-reported (CHQ-PF) versions of the Child Health Questionnaire to assess physical and psychosocial health (Landgraf et al., 1996).

##### Resource utilization

Regarding resource utilization, only one study assessed it through measuring coping. Fobian et al. (2020) used the Adolescent Coping Orientation for Problem Experiences (A-COPE; Patterson & McCubbin, 1983) to assess the strategies adolescents use to manage problems or difficult situations, including the use of services, medication, and social supports.

##### Other outcome measures

This review focused on assessment instruments, but it is important to note that some studies also used other types of outcome measures, such as symptom frequency, school attendance, and hospital readmissions (Byrne & Connon, 2023; Demirci & Sagaltici, 2021; Fobian et al., 2020; Kozlowska et al., 2018; Maxwell et al.,2023; Rajabalee et al., 2022; Stager et al., 2023). Moreover, one study (McFarlane et al., 2019) used a personalized Goal-Based Outcomes measure, which measures how much a person feels they have moved toward an identified goal (Law, 2015). While these measures were not the focus of this review, they could offer further insight into certain COMET domains.

#### Psychometric properties of assessment tools

None of the studies included in this review examined the psychometric properties of the assessment tools. To address this gap, the main author conducted targeted searches using the name of each measure alongside psychometric term of interest. The searches were guided by measurement domains outlined in the European Federation of Psychologists’ Association (EFPA) review model for evaluating psychological and educational tests (Evers et al., 2013). These domains include reliability (internal consistency, test–retest reliability, and interrater reliability) and validity (criterion and construct validity). In addition to this, evidence for factorial invariance was included. Factorial invariance is particularly important when examining outcome measures, as it evidences that the measure consistently captures the same construct across time and in different groups (Putnick & Bornstein, 2016). This review did not assess the methodological quality of these studies and the EFPA review model was used to guide the search for evidence related to the psychometric properties of the assessment tools (Table 2).

**Table 2.** Psychometric properties of identified assessment tools.

Assessment tool	References	Population	Reliability			Validity		
			Internal consistency	Test–retest	Interrater reliability	Criterion validity	Construct validity	Factorial invariance
AAQ-II	–	No pediatric data	No pediatric data	No pediatric data	N/A	No pediatric data	No pediatric data	No pediatric data
A-COPE	Blount et al., 2008; Patterson & McCubbin, 1987	Community and clinical samples	α=.50–.76	No data	N/A	✓ (concurrent and predictive)	No data	No data
ADES	Correia-Santos et al., 2022; Howard et al., 2022; Keck Seeley et al., 2004	Community and clinical samples	α=.94	*r*=.77–.91	N/A	✓ (concurrent and predictive)	convergent validity not supported	✓
BASC self-report of personality	Reynolds & Kamphaus, 2004, 2015; Tan, 2007	Community and clinical samples	α=.60 s–.90 s	*r*=.70 s–.90 s	*Mdn*_teachers_=.53–.65 *Mdn*_parents_=.69 –.77	✓ (concurrent)	✓ (convergent)	No pediatric data
BDI-II	Basker et al., 2007; Keller et al., 2020; Osman et al., 2004	Clinical samples	α=.80–.96	*r*=.82	N/A	✓ (concurrent and predictive)	✓ (convergent and discriminant)	Partial
BPQ	–	No pediatric data	No pediatric data	No pediatric data	N/A	No pediatric data	No pediatric data	No pediatric data
BYI-II	Somerville et al., 2024; Stapleton et al., 2007	Community and clinical samples	α=.86–.96	*r*=.74–.93	N/A	✓ (concurrent and predictive)	✓ (convergent and discriminant)	No data found
CES-DC	Faulstich et al., 1986; Li et al., 2010; Zhao et al., 2024	Clinical sample	α=.82–.89	*r*_children_=.12 and *r*_adolescents_=.69	N/A	✓ (concurrent)	✓ (convergent and discriminant)	✓
CGAS	Bird et al., 1987; Lundh et al., 2016; Shaffer et al., 1983	Clinical samples	N/A	*r*=.69–.95	ICC=.84	✓ (concurrent and predictive)	✓ (convergent and discriminant)	N/A
CHQ	Hullmann et al., 2011; Raat et al., 2002; Veronese & Pepe, 2018	Community and clinical samples	α_parent-report_=.39–.96 and α_self-report_=.69–.92	ICC_parent-report_=.03–0.79	No data	No data	✓ (convergent for parent form)	Partial
COPM	Cusick et al., 2007	Children with disabilities	α=.86	No pediatric data	N/A	No pediatric data	No pediatric data	No data
CSSI-24	Essau et al., 2013; Garber et al., 1991; Meesters et al., 2003; Walker et al., 2009	Community and clinical samples (somatic symptoms without a physical condition)	α=.88–.92	*r*=.50–.66	Rho=.50 *r*=.44	✓ (concurrent and predictive)	✓ (convergent and discriminant)	No data
DASS	Mellor et al., 2015; Patrick et al., 2010	Community sample	α=.83–.96	No pediatric data	N/A	No pediatric data	✓ (convergent)	✓
FDI	Solé et al., 2019; Walker & Greene, 1991	Community and abdominal pain sample	α=.75–.92	*r*=.63–.80	No data	✓ (concurrent and predictive)	✓ (convergent and discriminant)	No data
LEFS	Chua et al., 2024	Lower limb conditions	α=.97	No pediatric data	N/A	✓ (concurrent)	✓ (convergent)	No data
OMNI-RPE	Gammon et al., 2016; Pfeiffer et al., 2002	Community sample	N/A	ICC=.95 (between trials)	N/A	✓ (concurrent)	No pediatric data	N/A
PCS-C	Crombez et al., 2003; Parkerson et al., 2013	Community sample and chronic pain conditions	α=.68–.87	No data	N/A	✓ (predictive)	✓ (convergent)	✓
PedsQL	Bastiaansen et al., 2004; Desai et al., 2014; Limbers et al., 2008; Scarpelli et al., 2008; Varni et al., 2001	Community and clinical samples (neurological conditions)	α=.80–.88	*r* = 0.69–0.90	No data	✓ (predictive)	✓ (convergent and discriminant)	✓
RAHC-GAF	Dossetor et al., 1996	Clinical sample	N/A	No data	*r*=.67–.78 (between clinicians)	✓ (concurrent)	No data	N/A
RCADS	Cervin et al., 2022; Chorpita et al., 2000, 2005; Ebesutani et al., 2010, 2015	Clinical samples	α_parent-report_=.81–.95 α_self-report_=.71–.85	*r*_parent-report_=0.79–0.93 *r*_self-report_=0.65–0.80	No data found	✓ (concurrent for self-report)	✓ (convergent and discriminant)	✓
SAS	Setyowati et al., 2019	Community sample	α=.69	No data	N/A	No data	✓ (convergent)	No data
SCAS	Arendt et al., 2014; Galán-Luque et al., 2025	Community and clinical samples	α=.42–.97	*r* = 0.60–0.91	No data	No data	✓ (convergent and discriminant)	✓
SDQ	Mieloo et al., 2012; Murray et al., 2022; Stevanovic et al., 2015; Vugteveen et al., 2021; Wolpert et al., 2015	Community and clinical samples	α_parent-report_=.49–.77 α_self-report_=.17–0.87	*r* = 0.45–0.70	ICC = 0.21–0.44 (parent and teacher form)	✓ (concurrent and predictive)	✓ (convergent and discriminant)	Partial invariance
UEFI	–	No pediatric data	No pediatric data	No pediatric data	N/A	No pediatric data	No pediatric data	No data
WSAS-Y	Jassi et al., 2020; Koc et al., 2025	Community and clinical samples	α=.82–.90	*r* = .69–.80	No data	✓ (concurrent)	✓ (convergent and discriminant)	No data
YGTSS	Storch et al., 2005; Walkup et al., 1992; Wen et al., 2021	Tic disorders, excluding functional tics	α=.92–.94	ICC=.77–.90	Rho=.93	✓ (concurrent)	✓ (convergent and discriminant)	✓

*Note*. α=Cronbach’s Alpha; ICC=interclass correlation; Mdn=median reliability; *r*=Pearson’s correlation; Rho=Spearman’s rank coefficient.

A community sample refers to children from nonclinical settings, while a clinical sample refers to children in clinical settings or with a confirmed clinical diagnosis.

None of the identified tools were specifically designed to measure outcomes in FND, and most studies on their psychometric properties did not include pediatric FND samples. Therefore, these tools have not been validated for this population. Identified instruments have been discussed between the authors in order to create a list of recommended tools (Table 3). In providing recommendations for COMET domains, the following criteria were considered: (a) whether the instrument has been validated in a pediatric population; (b) evidence of criterion validity in the literature; (c) evidence of construct validity in the literature; (d) when applicable, evidence of internal reliability; (e) evidence of test–retest reliability; (f) evidence of interrater reliability, and (g) evidence of factorial invariance.

**Table 3.** Recommendations for assessment tools for outcome measurement in FND.

Domain	Assessment tool
Core FND symptoms	YGTSS (for functional tics)
Mental health	Implementing clinician-rated measures alongside self- and parent-reports Using tools that capture physical and cognitive aspects of mental health More research is needed to determine adequate measures in this population
Other symptoms	CSSI-24
Life impact	Quality of life PedsQL Functional independence WSAS-Y for social functioning FDI for general functioning CGAS for clinician ratings
Resources	More research is needed to determine adequate measures

*Note*. FND=functional neurological disorder; CGAS=Children’s Global Assessment Scale; CSSI-24=Children’s Somatic Symptoms Inventory; FDI=Functional Disability Inventory; PedsQL=Pediatric Quality of Life Inventory; WSAS-Y=Work and Social Adjustment Scale—Youth Version; YGTSS=Yale Global Tic Severity Scale.

## Discussion

This review identifies instruments used for measuring outcomes of psychological interventions in pediatric FND, outlines outcome domains, and offers recommendations for future research and practice. The outcome domains that were covered in the included studies were functional independence and quality of life, core symptoms, mental health, other symptoms, and coping.

### Outcome domains and assessment tools in psychological interventions

None of the tools identified in this study are validated for pediatric FND populations. Since measurement properties can vary across groups, it is essential to assess these tools within the target population (Terwee et al., 2007). In the absence of validated FND-specific tools, using reliable, validated pediatric tools is recommended (Table 3).

#### Core FND symptoms

The YGTSS is the only tool identified for measuring core symptoms of tics. It has evidence of being valid and reliable, and is easy to administer, capturing the level of impairment in daily life. However, this tool is limited to assessing tics and is not suitable for measuring a full range of FND symptoms. Furthermore, there is currently no evidence supporting its validity and reliability with functional tics.

There is a noticeable lack of FND-specific outcome measures that can fully capture the broad spectrum of FND symptoms in adults or children (Pick et al., 2020). A systematic review conducted by Pick et al. (2020) identified two tools designed to assess various FND symptoms in children: the Conversion Disorder Scale (CDS; Sarfraz & Ijaz, 2014) and its updated version (CDS-R; Ijaz et al., 2017). The authors of these measures reported evidence of acceptable internal consistency, concurrent validity, and the ability to differentiate between healthy children and those with FND. However, further evidence regarding their psychometric properties is currently limited. To the authors’ knowledge, no other tools specifically designed for pediatric FND currently exist. The diverse symptomatology of FND makes it unclear whether a single measure could adequately capture all its variations. In the absence of tools for measuring core symptoms, clinicians, and researchers may need to rely on alternative data sources, such as symptom journaling.

#### Mental health

Most studies assessed anxiety and depression, though some tools lacked strong reliability or validity evidence for pediatric use. The RCADS stands out as a widely used measure of anxiety and depression with good psychometric properties, including strong test–retest reliability, validity, and factorial invariance. One of the benefits of the RCADS is that it includes items that assess physical symptoms of anxiety, such as “When I have a problem, I feel shaky,” which may be more accessible for children with FND to respond to. However, a study conducted by McFarlane et al. (2019) showed that in many cases RCADS self-reported and parent-reported scores did not correlate with a clinical diagnosis and did not detect reliable change posttreatment. This suggests that, despite children with FND displaying symptoms of anxiety or depression, the severity of these symptoms was not reflected by the scores. If these tools fail to capture the symptoms they are designed to assess, it becomes difficult to determine whether any meaningful change has occurred.

A range of other mental health measures have been used, but all present limitations in the context of pediatric FND. Some studies relied solely on self-reports, which may be problematic given the difficulties this population might face in reporting emotional symptoms. Parent and teacher report tools such as the BASC detected posttreatment changes mainly in somatic symptoms, but not in depression or anxiety, suggesting limited sensitivity to emotional functioning changes. Cognitive processes, such as pain catastrophizing, were rarely assessed, and when they were, the available tools lacked comprehensive validation in FND populations. Most studies in this review overlooked cognitive factors such as illness perception, sense of control, and negative symptom expectations, which are common in FND and may influence symptom maintenance (Kozlowska et al., 2023).

Focusing on and measuring emotional functioning in treatment is important, as children with FND often face challenges in this area, including higher levels of anxiety, low mood, passive and avoidant coping, and submissiveness (Sawchuk & Buchhalter, 2015). However, these children might struggle to differentiate between physical and psychological symptoms (Gulpek et al., 2014), which might influence their responses on self-reported measures (Kozlowska et al., 2016). One study with children with severe FND found that self-reports often missed identifying distress or intervention needs (Kozlowska et al., 2016). Similarly, another study (McFarlane et al., 2019) found that traditional self- and parent-report ratings failed to capture emotional symptoms despite clinical signs. Attachment styles can also influence responses, as children with FND are more frequently classified within at-risk attachment groups (Kozlowska et al., 2011). Some children tend to habitually suppress negative emotions, presenting falsely positive responses to manage attachment figures’ reactions (Crittenden, 1999). This strategy of inhibiting negative affect can impact how these children communicate with others, including healthcare professionals, and lead to biased self-reports (Kozlowska et al, 2016). There is also a possibility that families underreport psychological difficulties, due to stigma associated with FND and fears of not getting adequate medical treatments (McFarlane et al., 2019); Kozlowska et al., 2021).

These findings point to discrepancies between clinical observations of emotional functioning and the difficulties captured with measures in this domain. This might be due to the limitations of the tools themselves, but also the challenges often seen in this population, who may struggle with recognizing and expressing internal states, and stigma. Therefore, current evidence makes it difficult to recommend mental health assessment tools for children with FND. More research is needed to identify suitable tools, especially for clinical practice, as there is some evidence suggesting self-report mental health assessment tools might be more useful in research settings and group-level analyses (Kozlowska et al., 2016).

#### Other symptoms

Other symptoms measured in the identified studies included somatic symptoms, body stress reactions, and perceived exertion. These are important outcomes for this population as they are prevalent and highly impactful. The OMNI-RPE, while not a typical questionnaire, is a scale used to measure perceived effort or exertion following a task. Studies indicate that children with FND tend to perceive tasks as more exhausting compared to control groups (Barak et al., 2022; Landa et al., 2023), making this an important outcome domain; however, it lacks evidence of predictive and construct validity.

The CSSI-24 is the only measure validated in samples that include children with symptoms without a physical cause. It shows strong psychometric properties, is brief, and easy to administer. Another advantage is that it has a parent form, though it was not used in the included studies. For researchers or clinicians seeking parent and teacher ratings, the BASC somatic scale can be considered, which also has an updated third version published (Reynolds & Kamphaus, 2015). Nonetheless, future studies need to evaluate its psychometric properties within FND samples.

A small number of studies included measures of other symptoms, despite a range of difficulties that are commonly reported by children with FND. Considering the discussed problems with mental health tools, including measures of other symptoms, could provide insights into the potential impact of interventions that may be otherwise missed.

#### Life impact

Quality of life and functional independence are essential for assessing outcomes in FND, given the debilitating and burdensome nature of many FND symptoms. Moreover, such measures of impact of symptoms are likely relevant across numerous symptom types in FND. A variety of assessment tools were used in this domain, many of which lack adequate psychometric validation.

Health-related quality of life was measured using the PedsQL and CHQ. The PedsQL has demonstrated good psychometric properties and has been validated with neurological samples. Although more research is needed, the PedsQL shows promise as a self-report measure for this population. It has a parent-report version, which allows gathering information from parent’s perspective. The CHQ also has strong psychometric properties, though concerns exist about test–retest reliability for physical functioning and social physical functioning subscales, which are likely relevant to FND.

Various functional independence measures were used in the studies. The WSAS-Y is a very brief and simple tool with strong psychometric properties that captures social functioning, including school. This can provide important insights and inform intervention planning, as FND symptoms often affect how children are doing academically and in peer relationships (Borner & Laptook, 2024). The FDI is another measure that showed good psychometric properties. It is validated for patients experiencing chronic pain, which is often seen in FND cases (Yong et al., 2023). Both WSAS-Y and FDI showed posttreatment change in reviewed studies and include parent forms. For clinician ratings, the CGAS demonstrated good psychometric properties, with strong test–retest and inter-rater reliability. In a study by McFarlane et al. (2019), which used various self-reports of functioning and mental health, the CGAS was the only tool that detected posttreatment change.

This review identified several tools with promising properties in this domain, offering options for clinician-rated, parent-report, or self-report assessment. These measures could provide a more holistic indication of change posttreatment that is not symptom-focused. However, all these measures require validation in FND samples and further assessment of their factorial invariance.

#### Resources

Measuring healthcare or social resources with assessment tools was largely neglected. The A-COPE, the only questionnaire used in this domain, lacks evidence of test–retest reliability and construct validity. Research suggests that children with FND are more likely to engage in maladaptive coping strategies (Sawchuk & Buchhalter, 2015), making this an important area for both intervention and outcome measurement.

#### Adverse events and mortality

As outlined above, domains of adverse events and mortality were excluded. However, assessment instruments identified in this review could be used to measure the potential worsening of symptoms and functioning, emergence of new symptoms, or suicidality. Research indicates that measurement of adverse events in child psychological interventions is largely neglected and inconsistent. Most intervention studies do not report adverse events, and when they do, they often lack adequate information about impact and severity (Lodewyk et al., 2023). However, research on youth shows that some report negative experiences during psychological treatment (Watson et al., 2023). For example, a qualitative study conducted with children with FND found that some children report seeing psychological support as “blaming” (Staton et al., 2024). Recommendations from the literature state the need for a comprehensive and consistent approach to monitoring negative outcomes (Lodewyk et al., 2023). Overall, there is a need for research focusing on the measurement of adverse events of psychological interventions in this population.

### Recommendations

Taken together, these findings highlight several limitations in measuring outcomes in children with FND in the current literature. Most studies relied on using tools developed for other conditions and populations (e.g., depression). While measuring different domains is important in a complex condition as FND, this also points to a lack of FND-specific measures. Moreover, there is a significant lack of validation studies in children and adolescents with FND and existing tools should undergo rigorous validation within this population. Until then, tools with proven reliability and validity in broader pediatric samples should be used. Future studies with children with FND should aim to incorporate data on the measurement properties of the used questionnaires, and in that way contribute to the evidence for these tools in pediatric FND populations (Pick et al., 2020). Most studies did not acknowledge the lack of validation for FND and provided limited information about the tools. Researchers should explicitly acknowledge this limitation and justify their choice of outcome measures.

This review identified tools with good psychometric properties, however, the lack of validation in FND samples raises a question about their suitability for this population, particularly when assessing mental health. Current research indicates that self-reports may not fully capture emotional difficulties in this population (Kozlowska et al., 2016; McFarlane et al., 2019). Lower baseline scores at the start of intervention, potentially influenced by difficulties in recognizing emotions, stigma, or response inhibition styles, could make it more challenging to detect meaningful change. Of course, there is a possibility that interventions failed to address emotional functioning adequately, or that FND was misdiagnosed when there was an organic disorder. However, this seems less likely given the strict diagnostic and defined psychological interventions criteria used in the included studies. Measures with limited psychometric support can hinder making reliable conclusions about intervention efficacy. If a tool does not adequately and reliably capture the construct of interest, any observed change (or lack of change) may reflect measurement error rather than treatment effects.

Including clinician-rated measures with self- and parent-reports could help, given the potential for inhibition of negative responses in FND. Additionally, items focusing on somatic symptoms may resonate better with these families. Adjusting the language of existing questionnaires might make them more accessible, shifting away from psychiatric terms to language that may feel more relatable (e.g., asking if they feel tense rather than anxious) (Kozlowska et al., 2016). Focusing on life impact measures may also be a helpful and more meaningful strategy, as they encompass various domains often affected by FND, such as level of independence, school, and relationships. Studies utilizing both mental health and life impact measures frequently found posttreatment changes in life impact domains, whereas changes in mental health measures were less commonly observed. Future research should explore these considerations, as mental health is vital in FND presentation. Finally, assessment tools should be combined with additional methods, such as family interviews, reported symptoms, and school attendance. FND is a complex condition that can present differently in each child. Given this diversity, the question arises as to whether integrating individualized outcome measures, such as goal-based outcomes, alongside standardized assessment tools across relevant domains, could provide a more comprehensive evaluation of treatment effects. These personalized approaches could be guided by clinical judgment and what the child and their family consider most meaningful. However, further research is needed to explore the potential benefits of integrating both standardized and individualized outcome measures with this population.

### Limitations

Most of the included studies were conducted in high-income countries, and all articles were written in English. While cross-cultural research is still limited, some studies suggest that symptom patterns of FND in developing countries differ from those in Western countries (Ijaz et al., 2017). Moreover, many behaviors or experiences meant to be captured by assessment instruments can have different conceptual and operational definitions shaped by cultural differences (Zhao et al., 2024). This raises the question of whether assessment instruments identified in this review would demonstrate consistent psychometric properties across different cultural and linguistic contexts. Further psychometric evaluation of these instruments across diverse FND populations is needed.

This review focused on treatments involving psychological interventions, which are an important, but not the only component of the biopsychosocial approach. Other interventions, such as physical treatments or pharmacological approaches, were not captured in this review. Additionally, this review concentrated on assessment tools, and certain aspects of outcome domains, such as symptom frequency, monitoring return to school, or readmissions, were not included. Future research could explore a wider range of outcome measures in pediatric FND. As research in this area continues to grow, future studies could delve deeper into psychometric properties. In line with recommended guidelines, it would be beneficial to assess not only the characteristics of the questionnaires themselves but also the quality of the design and methodology used in their development (Mokkink et al., 2018; Prinsen et al., 2016; Terwee et al., 2007).

Finally, in examining the psychometric properties of assessment tools, inconsistencies were found in how authors defined and evaluated various forms of validity. Accessing information on certain questionnaires was also challenging due to publisher restrictions. Such limitations might create barriers for researchers and clinicians who are looking to make more informed decisions about which instruments to use. Additionally, some treatment studies that were examined in the process of this review did not describe the diagnostic process for FND, such as the criteria that was used or who was involved in the process. It would be important to provide a clear description of the diagnostic process in order to enhance the transparency and quality of future research.

## Conclusion

While this review has begun to explore outcome domains for pediatric FND, such as functional independence and quality of life, mental health, physical symptoms and other symptoms, and coping, it is only a starting point. Continued research and collaboration among FND experts are necessary to build on this work and establish consensus for measuring these domains. It is challenging to recommend FND-specific measures due to the limited use and lack of validation. Based on the current evidence, the recommendation is to rely on the assessment of relevant outcome domains with existing tools that are valid and reliable in pediatric populations. Moving forward, research should prioritize validating these measures within FND populations.

## Supplementary Material

jsaf071_Supplementary_Data
