# Supplementary material for: Systematic review of assessment instruments measuring outcomes in psychological interventions for pediatric functional neurological disorders
Source: J Pediatr Psychol. 2025 Aug 25;50(11):1061–77. doi: 10.1093/jpepsy/jsaf071 (PMC12633852; doi:10.1093/jpepsy/jsaf071)
Supplement: jsaf071_Supplementary_Data [file jsaf071_supplementary_data.docx]

| Table S1. *Excluded studies* |  |
| --- | --- |
| Study reference | Reason for exclusion |
| Baker, G. A., Moore, P., & Appleton, R. E. (1995). Non-epileptic attack disorders in children and adolescents: a single case study. *Seizure*, *4*(4), 307–309. <https://doi.org/10.1016/s1059-1311(95)80009-3> | Validated questionnaire not used as an outcome measure. |
| Barak, S., Landa, J., Eisenstein, E., Gerner, M., Ravid Vulkan, T., Neeman-Verblun, E., & Silberg, T. (2024). Agreement and disagreement in pediatric functional neurological symptom disorders: Comparing patient reported outcome measures (PROMs) and clinician assessments. *Computational and structural biotechnology journal*, *24*, 350–361. <https://doi.org/10.1016/j.csbj.2024.04.045> | Validated questionnaire not used as an outcome measure. |
| Bolger, A., Collins, A., Michels, M., & Pruitt, D. (2018). Characteristics and Outcomes of Children with Conversion Disorder Admitted to a Single Inpatient Rehabilitation Unit, A Retrospective Study. *PM & R: the journal of injury, function, and rehabilitation*, *10*(9), 910–916. <https://doi.org/10.1016/j.pmrj.2018.03.004> | Psychological intervention not clear. |
| Butz, C., Iske, C., Truba, N., & Trott, K. (2019). Treatment of Functional Gait Abnormality in a Rehabilitation Setting: Emphasizing the Physical Interventions for Treating the Whole Child. *Innovations in clinical neuroscience*, *16*(7-08), 18–21. | Unclear diagnosis not meeting criteria. |
| Deaton, A. V. (1998). Treating conversion disorders: Is a pediatric rehabilitation hospital the place? *Rehabilitation Psychology, 43*(1), 56–62. [https://doi.org/10.1037/0090-5550.43.1.56](https://psycnet.apa.org/doi/10.1037/0090-5550.43.1.56) | Measure not referenced. |
| Donohue, B., Thevenin, D. M., & Runyon, M. K. (1997). Behavioral treatment of conversion disorder in adolescence. A case example of Globus Hystericus. *Behavior modification*, *21*(2), 231–251. <https://doi.org/10.1177/01454455970212006> | Validated questionnaire not used as an outcome measure. |
| Duncan, M., Pearman, Z., Harrold, K., Warren, A., Evans, S., McAllister, E., Heyman, I., Shavel-Jessop, S., Murphy, T., & Liang, H. (2024). Evaluation of a psychoeducation group for children presenting with functional tic-like behaviours. *Clinical child psychology and psychiatry*, *29*(3), 1011–1025. <https://doi.org/10.1177/13591045241237829> | Psychological intervention not meeting criteria. Also, validated questionnaire not used as an outcome measure. |
| Flewelling, K. D., Koehler, A., Shaffer, J., & Dill, E. J. (2020). Medical and psychosocial outcomes of youth with psychogenic nonepileptic seizures: An observational study. *Epilepsy & behavior: E&B*, *112*, 107383. <https://doi.org/10.1016/j.yebeh.2020.107383> | Psychological intervention not meeting criteria. |
| Fredwall, M., Terry, D., Enciso, L., Burch, M. M., Trott, K., & Albert, D. V. F. (2021). Outcomes of children and adolescents 1 year after being seen in a multidisciplinary psychogenic nonepileptic seizures clinic. *Epilepsia*, *62*(10), 2528–2538. <https://doi.org/10.1111/epi.17031> | Validated questionnaire not used as an outcome measure. |
| Fredwall, M., Terry, D., Enciso, L., Burch, M. M., Trott, K., & Albert, D. V. F. (2021). Short-term outcomes in pediatric and adolescent patients with psychogenic nonepileptic events seen by telemedicine during the COVID-19 pandemic. *Epilepsy & behavior: E&B, 117*, 107739. <https://doi.org/10.1016/j.yebeh.2020.107739> | Psychological intervention not meeting criteria. |
| Gerner, M., Barak, S., Landa, J., & Eisenstein, E. (2016). Parent-child Communication-centered Rehabilitative Approach for Pediatric Functional Somatic Symptoms. *The Israel journal of psychiatry and related sciences*, *53*(2), 39–46. | Validated questionnaire not used as an outcome measure. |
| Goldstein, M., Madden, S., & Peters, L. (2013). The use of effective treatments: the case of an adolescent girl with anorexia nervosa in the context of a conversion disorder. *Clinical child psychology and psychiatry*, *18*(2), 214–223. <https://doi.org/10.1177/1359104512447313> | Dual diagnosis. Also, validated questionnaire not used as an outcome measure. |
| Gooch, J. L., Wolcott, R., & Speed, J. (1997). Behavioral management of conversion disorder in children. *Archives of physical medicine and rehabilitation*, *78*(3), 264–268. <https://doi.org/10.1016/s0003-9993(97)90031-9> | Validated questionnaire not used as an outcome measure. |
| Harwood, A., Shalev, A., Ben-Shaul, S., Meir, R., Kiansky, E., Horn, K., Deitcher, C., Dror, S., & Galili, E. (2018). Retreating From Life: The Boy Whose Body Experienced His Pain. *Clinical Case Studies, 17*(6), 406-424. <https://doi-org.elib.tcd.ie/10.1177/1534650118793969> | Diagnosis not meeting criteria. |
| Hoffman, R., Bibby, H., Bennett, D., Klineberg, E., Rushworth, A., & Towns, S. (2016). Family functioning as a protective factor in treating adolescents with complex medico-psychosocial presentations. *International journal of adolescent medicine and health*, *28*(4), 437–444. <https://doi.org/10.1515/ijamh-2015-0037> | Not clear if sample included children with FND diagnosis. |
| Kallesøe, K. H., Schröder, A., Jensen, J. S., Wicksell, R. K., & Rask, C. U. (2021). Group-based Acceptance and Commitment Therapy (AHEAD) for adolescents with multiple functional somatic syndromes: A randomised trial. *JCPP advances*, *1*(4), e12047. <https://doi.org/10.1002/jcv2.12047> | Not clear if sample included functional neurological disorders. |
| Kasia, K., Nicola, G., Stephen, S., & Blanche, S. (2021). Psychologically informed physiotherapy as part of a multidisciplinary rehabilitation program for children and adolescents with functional neurological disorder: Physical and mental health outcomes. *Journal of paediatrics and child health*, *57*(1), 73–79. <https://doi.org/10.1111/jpc.15122> | Physiotherapy primary intervention. |
| Khachane, Y., Kozlowska, K., Savage, B., McClure, G., Butler, G., Gray, N., Worth, A., Mihailovich, S., Perez, D. L., Helgeland, H., & Chrousos, G. P. (2019). Twisted in Pain: The Multidisciplinary Treatment Approach to Functional Dystonia. *Harvard review of psychiatry*, *27*(6), 359–381. <https://doi.org/10.1097/HRP.0000000000000237> | Validated questionnaire not used as an outcome measure. |
| Klonoff, E. A., & Moore, D. J. (1986). "Conversion reactions" in adolescents: a biofeedback-based operant approach. *Journal of behavior therapy and experimental psychiatry*, *17*(3), 179–184. <https://doi.org/10.1016/0005-7916(86)90024-8> | Validated questionnaire not used as an outcome measure. |
| Kozlowska, K., English, M., Savage, B., Chudleigh, C., Davies, F., Paull, M., Elliot, A., & Jenkins, A. (2013). Multimodal rehabilitation: A mind-body family-based intervention for children and adolescents impaired by medically unexplained symptoms. Part 2: Case studies and outcomes. American Journal of Family Therapy, 41(3), 212–231. <https://doi.org/10.1080/01926187.2012.677723> | Validated questionnaire not used as an outcome measure. |
| Kozlowska, K., Chudleigh, C., Savage, B., Hawkes, C., Scher, S., & Nunn, K. P. (2023). Evidence-Based Mind-Body Interventions for Children and Adolescents with Functional Neurological Disorder. *Harvard review of psychiatry*, *31*(2), 60–82. <https://doi.org/10.1097/HRP.0000000000000358> | Validated questionnaire not used as an outcome measure. |
| Lenart-Domka, E., & Pelc-Dymon, M. (2018). Multi-profile procedures for motor conversion disorders in children - a case report. Wieloprofilowe postępowanie w konwersyjnych zaburzeniach ruchu u dzieci – opis przypadku. *Psychiatria polska*, *52*(4), 685–695. <https://doi.org/10.12740/PP/OnlineFirst/43145> | Validated questionnaire not used as an outcome measure. |
| Leslie, S. A. (1988). Diagnosis and treatment of hysterical conversion reactions. *Archives of disease in childhood*, *63*(5), 506–511. <https://doi.org/10.1136/adc.63.5.506> | Validated questionnaire not used as an outcome measure. |
| Lock, J., & Giammona, A. (1999). Severe somatoform disorder in adolescence: A case series using a rehabilitation model for intervention. *Clinical Child Psychology and Psychiatry, 4*(3), 341–351. <https://doi.org/10.1177/1359104599004003005> | No FND diagnosis. |
| Malhi, P., Kumar, C., Singhi, P., & Sankhyan, N. (2021). Outcome of Conversion Symptoms in Children. *Indian journal of pediatrics*, *88*(4), 367–369. <https://doi.org/10.1007/s12098-020-03465-y> | Unclear psychological intervention. |
| Masia Warner, C., Reigada, L. C., Fisher, P. H., Saborsky, A. L., & Benkov, K. J. (2009). CBT for anxiety and associated somatic complaints in pediatric medical settings: an open pilot study. *Journal of clinical psychology in medical settings*, *16*(2), 169–177. <https://doi.org/10.1007/s10880-008-9143-6> | No FND diagnosis. |
| McKinlay, T., Kelly, J. A., & Collum, J. M. (1977). The multi‐modal treatment of conversion reactions in adolescence: A case study. *Journal of Clinical Child Psychology*, *6*(3), 66–68. <https://doi.org/10.1080/15374417709532788> | Validated questionnaire not used as an outcome measure. |
| Mooney, G., & Gurrister, T. (2004). Behavioral Treatment of Psychogenic Deafness: A Case Report. *Rehabilitation Psychology, 49*(3), 268–271. [https://doi.org/10.1037/0090-5550.49.3.268](https://psycnet.apa.org/doi/10.1037/0090-5550.49.3.268) | Validated questionnaire not used as an outcome measure. |
| Negash, A., Abera, M., Gruber-Frank, C., & Frank, R. (2015). An adolescent with significant emotional and medically unexplained complaints: case report and proposal of an intervention. *Child and adolescent psychiatry and mental health*, *9*, 48. <https://doi.org/10.1186/s13034-015-0080-5> | Validated questionnaire not used as an outcome measure. |
| Oberfield, R. A., Reuben, R. N., & Burkes, L. J. (1983). Interdisciplinary approach to conversion disorders in adolescent girls. *Psychosomatics*, *24*(11), 983–989. <https://doi.org/10.1016/S0033-3182(83)73123-3> | Validated questionnaire not used as an outcome measure. |
| Palermo, T. M., & Scher, M. S. (2001). Treatment of functional impairment in severe somatoform pain disorder: a case example. *Journal of pediatric psychology, 26*(7), 429–434. <https://doi.org/10.1093/jpepsy/26.7.429> | No FND diagnosis. |
| Parraga, H. C., & Kashani, J. H. (1981). Treatment approach in a child with hysterical seizures superimposed on partial complex seizures. *Canadian journal of psychiatry. Revue canadienne de psychiatrie*, *26*(2), 114–117. <https://doi.org/10.1177/070674378102600209> | Validated questionnaire not used as an outcome measure. |
| Pop-Jordanova, N., Zorcec, T., & Demerdzieva, A. (2005). Electrodermal biofeedback in treating psychogenic nonepileptic seizures. *Prilozi*, *26*(2), 43–51. | Validated questionnaire not used as an outcome measure. |
| Pop-Jordanova, N., & Zorcec, T. (2016). Somatoform Disorders - A Pediatric Experience. *Prilozi (Makedonska akademija na naukite i umetnostite. Oddelenie za medicinski nauki)*, *37*(2-3), 55–62. <https://doi.org/10.1515/prilozi-2016-0017> | Validated questionnaire not used as an outcome measure. |
| Spierings, C., Poels, P. J., Sijben, N., Gabreëls, F. J., & Renier, W. O. (1990). Conversion disorders in childhood: a retrospective follow-up study of 84 inpatients. *Developmental medicine and child neurology, 32*(10), 865–871. <https://doi.org/10.1111/j.1469-8749.1990.tb08098.x> | Validated questionnaire not used as an outcome measure. |
| Stager, L., Szaflarski, J. P., & Fobian, A. D. (2021). One-year follow-up of treatment outcomes and patient opinions of Retraining and Control Therapy (ReACT) for pediatric functional seizures. *Epilepsy & behavior reports*, *16*, 100503. <https://doi.org/10.1016/j.ebr.2021.100503> | Validated questionnaire not used as an outcome measure. |
| Szyndler, J. E., Towns, S., Hoffman, R. C., & Bennett, D. L. (2003). Clinical assessment, management and outcomes of a group of adolescents presenting with complex medico-psychosocial conditions. *Annals of the Academy of Medicine, Singapore*, *32*(1), 51–57. | Validated questionnaire not used as an outcome measure. |
| Terry, D., Enciso, L., Trott, K., Burch, M. M., & Albert, D. V. F. (2020). Outcomes in Children and Adolescents with Psychogenic Nonepileptic Events Using a Multidisciplinary Clinic Approach. *Journal of child neurology, 35*(13), 918–923. <https://doi.org/10.1177/0883073820939400> | Validated questionnaire not used as an outcome measure. |
| Turgay, A. (1990). Treatment outcome for children and adolescents with conversion disorder. *Canadian journal of psychiatry. Revue canadienne de psychiatrie*, *35*(7), 585–589. <https://doi.org/10.1177/070674379003500704> | Validated questionnaire not used as an outcome measure. |
| Wilkinson-Smith, A., Greenberg, B., & Keech, A. (2020). The princess and the *p*-value: A case report of suspected autoimmune encephalitis and functional neurological disorder in a pediatric patient. *Applied neuropsychology. Child*, *9*(1), 13–20. <https://doi.org/10.1080/21622965.2018.1501373> | No clear psychological intervention besides assessment. Outcome measures only reported for pharmacological treatment. |

| Table S2. *Details of JBI Critical Appraisal Criteria and Assessment for included case studies* | | | | | |
| --- | --- | --- | --- | --- | --- |
| Study | Byrne & Connon, 2023 | Demirci & Sagaltici, 2021 | Kempert, 2021 | Nasiri et al., 2015 | Rajabalee et al., 2022 |
| Were patient’s demographic characteristics clearly described? | 1 | 1 | 1 | 1 | 1 |
| Was the patient’s history clearly described and presented as a timeline? | 1 | 1 | 1 | 1 | 1 |
| Was the current clinical condition of the patient on presentation clearly described? | 1 | 1 | 1 | 0 | 1 |
| Were diagnostic tests or assessment methods and the results clearly described? | 1 | 1 | 1 | 0 | 1 |
| Was the intervention(s) or treatment procedure(s) clearly described? | 1 | 1 | 1 | 0 | 1 |
| Was the post-intervention clinical condition clearly described? | 1 | 1 | 1 | 1 | 1 |
| Were adverse events (harms) or unanticipated events identified and described? | 0 | 0 | 0 | 0 | 0 |
| Does the case report provide takeaway lessons? | 1 | 1 | 1 | 1 | 1 |
| Overall appraisal | 7/8 | 7/8 | 7/8 | 4/8 | 7/8 |

| Table S3. *Details of JBI Critical Appraisal Criteria and Assessment for included case series* | | | |
| --- | --- | --- | --- |
| Study | Maxwell et al., 2023 | McFarlane et al., 2019 | Robinson et al., 2020 |
| Were there clear criteria for inclusion in the case series? | 1 | 1 | 1 |
| Was the condition measured in a standard, reliable way for all participants included in the case series? | 1 | 1 | 1 |
| Were valid methods used for identification of the condition for all participants included in the case series? | 1 | 1 | 1 |
| Did the case series have consecutive inclusion of participants? | 1 | 1 | 1 |
| Did the case series have complete inclusion of participants? | 1 | 0 | 0 |
| Was there clear reporting of the demographics of the participants in the study? | 1 | 1 | 1 |
| Was there clear reporting of clinical information of the participants? | 1 | 1 | 1 |
| Were the outcomes or follow up results of cases clearly reported? | 1 | 1 | 1 |
| Was there clear reporting of the presenting site(s)/clinic(s) demographic information? | 1 | 1 | 1 |
| Was statistical analysis appropriate? | 1 | 1 | 1 |
| Overall appraisal | 10/10 | 9/10 | 9/10 |

| Table S4. *Details of JBI Critical Appraisal Criteria and Assessment for included non-controlled pre- and post-studies* | | | | | | | |
| --- | --- | --- | --- | --- | --- | --- | --- |
| Study | Barak et al., 2022 | Howlett et al., 2022 | Klineberg et al., 2014 | Kozlowska et al., 2018 | Landa et al., 2023 | Scheurich et al., 2024 | Stager et al., 2023 |
| Is it clear in the study what is the “cause” and what is the “effect” (i.e. there is no confusion about which variable comes first)? | 1 | 1 | 1 | 1 | 1 | 1 | 1 |
| Was there a control group? | 0 | 0 | 0 | 1 | 1 | 0 | 0 |
| Were participants included in any comparisons similar? | N/a | N/a | N/a | 1 | 1 | N/a | N/a |
| Were the participants included in any comparisons receiving similar treatment/care, other than the exposure or intervention of interest? | N/a | N/a | N/a | 0 | 1 | N/a | N/a |
| Were there multiple measurements of the outcome, both pre and post the intervention/exposure? | 1 | 1 | 1 | 1 | 1 | 1 | 1 |
| Were the outcomes of participants included in any comparisons measured in the same way? | 1 | 1 | 1 | 1 | 1 | 1 | 1 |
| Were outcomes measured in a reliable way? | 1 | 1 | 1 | 1 | 1 | 1 | 1 |
| Was follow-up complete and if not, were differences between groups in terms of their follow-up adequately described and analyzed? | 1 | 0 | 0 | 1 | 0 | 0 | 0 |
| Was appropriate statistical analysis used? | 1 | 1 | 1 | 1 | 1 | 1 | 1 |
| Overall appraisal | 6/7 | 5/7 | 5/7 | 8/9 | 8/9 | 5/7 | 5/7 |

| Table S5. *Details of JBI Critical Appraisal Criteria and Assessment for the included RCT* | |
| --- | --- |
| Study | Fobian et al., 2020 |
| Was true randomization used for assignment of participants to treatment groups? | 1 |
| Was allocation to treatment groups concealed? | 1 |
| Were treatment groups similar at the baseline? | 1 |
| Were participants blind to treatment assignment? | 0 |
| Were those delivering the treatment blind to treatment assignment? | 0 |
| Were treatment groups treated identically other than the intervention of interest? | 0 |
| Were outcome assessors blind to treatment assignment? | 1 |
| Were outcomes measured in the same way for treatment groups? | 1 |
| Were outcomes measured in a reliable way? | 1 |
| Was follow up complete and if not, were differences between groups in terms of their follow up adequately described and analysed? | 1 |
| Were participants analysed in the groups to which they were randomized? | 1 |
| Was appropriate statistical analysis used? | 1 |
| Was the trial design appropriate and any deviations from the standard RCT design (individual randomization, parallel groups) accounted for in the conduct and analysis of the trial? | 1 |
| Overall appraisal | 10/13 |
